# Revealing Hidden Dynamics of Hydrogel-Based Desalination with ^23^Na Nuclear Magnetic Resonance

**DOI:** 10.1021/acs.jpcc.6c02171

**Published:** 2026-06-04

**Authors:** Huijing Zou, Chengtong Zhang, Florin Teleanu, Amir Jangizehi, Sebastian Seiffert, Alexej Jerschow

**Affiliations:** † Department of Chemistry, 5894 New York University, 100 Washington Square East, New York 10003, New York, United States; ‡ ELI-NP, “Horia Hulubei” National Institute for Physics and Nuclear Engineering, 30 Reactorului Street, Bucharest-Magurele 077125, Ilfov, Romania; § Department of Chemistry, Johannes Gutenberg-Universität Mainz, Duesbergweg 10−14, D-55128 Mainz, Rhineland Palatinate, Germany

## Abstract

Today’s fast-progressing water scarcity demands new energy-efficient water desalination technologies. Polyelectrolyte hydrogels are promising materials for this application as they can absorb water from saline solutions and reject salts. This process relies on the electrostatic imbalance between the charged groups in the hydrogel and the surrounding solution. However, current state-of-the-art methods for assessing desalination performance are primarily based on ionic conductivity measurements, which lack phase specificity, atomic-level insight, or spatial resolution, limiting research into the underlying microscopic mechanisms. We investigated salt-ion interactions in sodium polyacrylate hydrogels using ^23^Na nuclear magnetic resonance (NMR) spectroscopy throughout the swelling process, which provides detailed microscopic information on the different compartments. ^23^Na magnetic resonance imaging (MRI) is shown to map Na^+^ ion distributions across different environments, while polarization lifetime measurements are shown to probe ion dynamic regimes. Relaxation-edited imaging revealed the effects of the temperature and competing salts on ion behavior across phases. Additionally, multiple-quantum-filtered experiments selectively detected and characterized slow-tumbling Na^+^ ions, showing the presence of two dynamically distinct sodium pools. These findings highlight the power of integrated ^23^Na NMR and MRI techniques for the in situ analysis of ion distribution and dynamics in hydrogels, providing a detailed tool for tuning and optimizing hydrogel desalination strategies.

## Graphical abstract

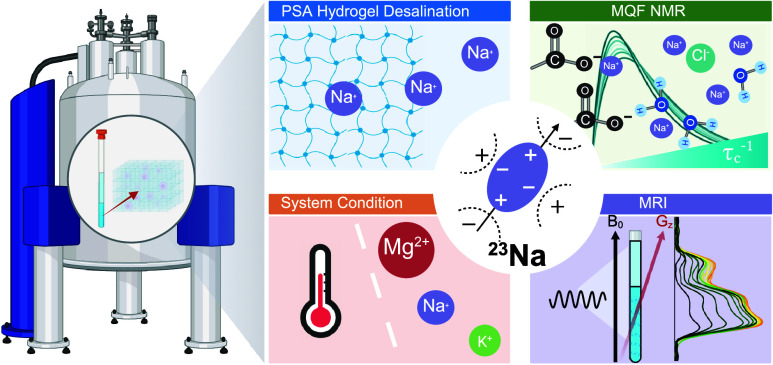

## 1 Introduction

Water scarcity is an ongoing global issue.^1,2^ In many geographical regions, desalination of seawater is considered a critical technology for supplying clean and safe water.^3,4^

Polyelectrolyte hydrogel desalination is a recently developed desalination method based on the Donnan effect, in which the fixed charge groups of the hydrogels create an electrostatic imbalance between the supernatant and the hydrogel phase.^5–9^ Common polyelectrolyte hydrogels include carboxylate-based and sulfonate-based backbones, such as poly­(sodium acrylate) (PSA), poly­(methacrylic acid) (PMA), and poly­(2-acrylamido-2-methyl-1-propanesulfonic acid) (PAMPS). Cross-linking improves materials’ performance and is generated with linkers such as N,*N*-methylene-bis-acrylamide (MBA).^5,10–12^ Among these hydrogels, PSA hydrogels exhibit high water absorbance, represent major industrial superabsorbent polymers, and can be produced at low cost.^13,14^ PSA hydrogels can absorb and retain large amounts of water while they selectively exclude salt ions, and fresh water can be recovered subsequently by heating or applying pressure.

Understanding the dynamics of salt ions and their distribution during the desalination process is important for evaluating the salt exclusion performance of hydrogels. Furthermore, these techniques are important for the optimization and tuning of hydrogel behavior. Current methods used for determining the salt rejection ratio focus mainly on measuring ionic conductivity through the swelling process and lack microscopic insights.^5,15–17^

As a potentially powerful alternative and complement to conductivity measurements, we investigate here the Na^+^ ion distribution and dynamics in the desalination system within both the supernatant and hydrogel phases using ^23^Na nuclear magnetic resonance spectroscopy and imaging methodologies.

NMR spectroscopy is a powerful tool for analyzing molecular dynamics within complex systems. Previous studies have used NMR spectroscopy to characterize the polymer structure based on the ^1^H and ^13^C NMR spectra^18–20^ and the swelling behavior of hydrogels based on ^1^H and ^23^Na diffusion rate analysis.^21–25^

In this study, ^23^Na NMR spectroscopy and imaging techniques were used to investigate the partitioning and dynamics of Na^+^ ions during the desalination process. Our results show that the spatial distribution of Na^+^ ions in the PSA hydrogel desalination system can be tracked through changes in temperature, swelling time, and salt composition. Because sodium nuclei have a spin quantum number of $\frac{3}{2}$ and an asymmetric nuclear charge distribution,^26,27^ the relaxation behavior of sodium nuclear spins is significantly dominated by the fluctuating electric field gradient (EFG) generated by surrounding atoms.^28^ The mobility of Na^+^ ions and their interactions with the hydrogel phase were characterized by monitoring changes in ^23^Na relaxation rates under different conditions.^29^ Furthermore, quadrupolar interactions between Na^+^ ions and the hydrogels were analyzed by using multiple-quantum-filtered (MQF) techniques. The MQF NMR experiment allows the detection of quadrupolar nuclei that exhibit large quadrupolar couplings in the slow-motion regime, characteristic of sodium ions that are strongly interacting with the fixed carboxylate groups.^30^ By combining MQF NMR with NMR imaging, we confirm the presence of slowly tumbling Na^+^ ions with fast relaxation rates within the PSA hydrogel desalination system and characterize their dynamics as a function of temperature. We also discuss the results of experiments with competing ions such as K^+^ and Mg^2+^.

## 2 Experimental Section

### 2.1 Materials

The PSA hydrogel is made of 100 mol % sodium acrylate with a cross-linking density of 10 mol % N,*N*-methylene-bis-acrylamide (MBA). The equilibrium swelling ratio (*Q*_eq_) in 17.1 mM NaCl solution is 20.09 g/g, which means that 1 g of dried PSA samples can absorb 20.09 g of 17.1 mM NaCl solution at room temperature. The PSA hydrogel and its properties were provided by Dr. Seiffert’s lab at Johannes Gutenberg University in Mainz. All salt solutions were prepared in D_2_O (99.9 atom % D, Sigma-Aldrich) at room temperature.

### 2.2 Preparation of Half/Full-Tube PSA Hydrogel Samples

The density of 17.1 mM NaCl in D_2_O was ∼1.0 g/mL, which is calculated by using the measured mass divided by volume. The amount of half-filled PSA in 500 μL NaCl was prepared using the half amount of PSA calculated from *Q*_eq_: $\frac{0.5\;mL\times \;1.0\;g/\mathrm{mL}}{2\,\;\times \;20.09\,\;g/g}=0.012$ g. In order to prepare a fully swollen PSA hydrogel sample, the amount of hydrogel added should be less than 0.012 g. The mass of PSA hydrogels added to the full-tube samples should be less than 0.024 g. In our experiment, we added 0.01 g of PSA hydrogels for half-tube samples in order to separate the samples into the supernatant phase and the gel region in the NMR detection region. And for the full-tube sample, 0.02 g of PSA hydrogels was added, which allows only the gel region to be shown in the detection region. Both samples were in the range of the mass calculated from. To ensure the hydrogel samples were fully equilibrated and ready for testing, we prepared all samples in the Sigma-Aldrich 5 mm diameter NMR tube and left the samples at room temperature for a minimum of 1 week to complete equilibrium before any measurement, except for the real-time measurements.

### 2.3 Preparation of Multisalt Solutions

Furthermore, the multisalt solution was prepared by adding KCl or MgCl_2_ as the competing salt to the NaCl solution. With a controlled concentration, the concentration of competing salts is the same as the concentration of NaCl (17.1 mM). Another group of samples was controlled by adjusting the total ionic strength of the solution to 17.1 mM. The solutions were prepared by mixing the same amount of NaCl with different amounts of KCl or MgCl_2_ in a total solution volume of 500 μL (Table S1). The total ionic strength (*I*) was calculated based on the formula:^31^ $I=\frac{1}{2}\sum_{i}c_{i}z_{i}^{2}$ , where *c*_*i*_ is the molar concentration of ion *i* and *z*_*i*_ is the charge number of ion *i*. Samples in multisalt solutions were prepared as shown in Table 1.

**Table 1:** Sample Preparation of Controlled Ionic Strength Experiments

sample	[NaCl] (mM)	[KCl] (mM)	[MgCl_2_] (mM)
in NaCl	17.1	0	0
in NaCl+KCl	10.95	5.69	0
in NaCl+MgCl2	10.95	0	2.03

### 2.4 Instrumentation

The ^23^Na NMR experiments were performed on a Bruker Avance III 9.4 T NMR spectrometer with a 5 mm BBFO probe and a GREAT 3/10 XYZ gradient amplifier. The maximum gradient strengths are 50 G/cm in the *X* and *Y* directions and 67 G/cm in the *Z* direction. The ^23^Na measurements were performed at 105.84 MHz. The temperature from 298 to 308 K was calibrated using methanol, while the temperature above 308 K was calibrated using ethylene glycol with the AU-calculator program.

### 2.5 Nonimaging Measurements

1D ^23^Na NMR spectra were acquired using the standard Bruker single pulse acquisition (1D zg). The 90° pulses and the offset of the transmitter frequency were optimized prior to each measurement. In ^23^Na NMR measurements, the saturation recovery pulse sequence (T1SR) was used for the longitudinal relaxation time (*T*_1_) measurement, and the Carr–Purcell–Meiboom–Gill (CPMG) pulse sequence was applied for the transverse relaxation time (*T*_2_) measurement. The *R*_1_ and *R*_2_ rates for ^23^Na were calculated from the reciprocal of the relaxation time and were obtained by fitting the experiment signal integrals (*S*) to the exponential functions derived from the Bloch equation:^28^ *S*(τ_1_) = *S*_0_(1–e^–*R*_1_τ_1_^) and *S*(τ_2_) = *S*_0_e^–*R*_2_τ_2_^, respectively. Here τ is the relaxation evolution time, which corresponds to the variable delay in *R*_1_ measurements and the transverse evolution time in *R*_2_ measurements. For lifetime measurements using imaging techniques, an additional offset was applied for baseline correction (See Section I in the Supporting Information).

### 2.6 Imaging Measurements

The Bruker standard 1D gradient echo shimming pulse sequence (1D imgegp) was used for the 1D ^23^Na NMR imaging experiments. This pulse sequence contains a 90° excitation pulse and a gradient echo. The gradient strength along the z direction (G_*z*_) was set at 2.08 G/cm (3.11%) and −4.52 G/cm (−6.74%), where the negative sign indicated a reversal of the gradient polarity. The acquisition settings of the 1D imaging profile include the FID size of 512 data points, the spectral width of 39682.5 Hz, and a total of 128 scans. The total duration of the gradient echo is 0.007 s. The relaxation-edited imaging experiments were performed using the modified saturation recovery pulse sequence and the modified CPMG pulse sequence, showing an additional gradient echo for detection. The acquisition settings of the FID size and the spectral width were the same as those used in the 1D NMR imaging experiments. Relaxation-edited imaging experiments were performed with 32 scans at temperatures from 298 to 318 K. The recycle delays applied in both nonimaging and imaging measurements were set to 5 times the *T*_1_ of the NaCl solution to ensure a full longitudinal magnetization recovery. And the recycle delays were adjusted accordingly as the temperature increased.

## 3 Results and Discussion

### 3.1 1D NMR and Imaging Characterization

As a first step, we show the spectral signatures of ^23^Na nuclei in the PSA-based prepared hydrogels. To compare spectroscopic data with imaging data, we compared samples prepared as either half-filled hydrogel tubes (prefix “h”) or full tubes (prefix “f”). The half-filled hydrogel tubes contain a swollen hydrogel region and a supernatant region of roughly equal extension within the detectable volume. Using one-dimensional NMR imaging techniques along the long axis of the NMR tube, we can clearly distinguish the hydrogel and the supernatant regions. To prove the validity of the imaging approach, we compared spectroscopy data of full tubes with the imaging approach. A saline solution is used for reference.

Sample preparation and NMR acquisition protocols and parameters are described in the Experimental Section and Sections I and II in the Supporting Information. Figure 1A shows the 1D ^23^Na NMR spectra of a 17.1 mM NaCl solution, the half-filled PSA in D_2_O (h-PSA), and the half-filled PSA in NaCl solution in D_2_O (h-PSA-NaCl). The measured full widths at half-maximum (FWHM) of the observed peaks were 6.89, 39.58, and 19.46 Hz, respectively. The larger ^23^Na FWHM was shown for the sample of PSA in D_2_O because a larger fraction of Na^+^ ions remained strongly bound with the hydrogel phase, which provided a slower motion and a larger residual quadrupolar interaction. For the h-PSA-NaCl sample, there were more free Na^+^ ions in the system, which averaged the quadrupolar interaction, therefore resulting in a narrower FWHM.

**Figure 1:**
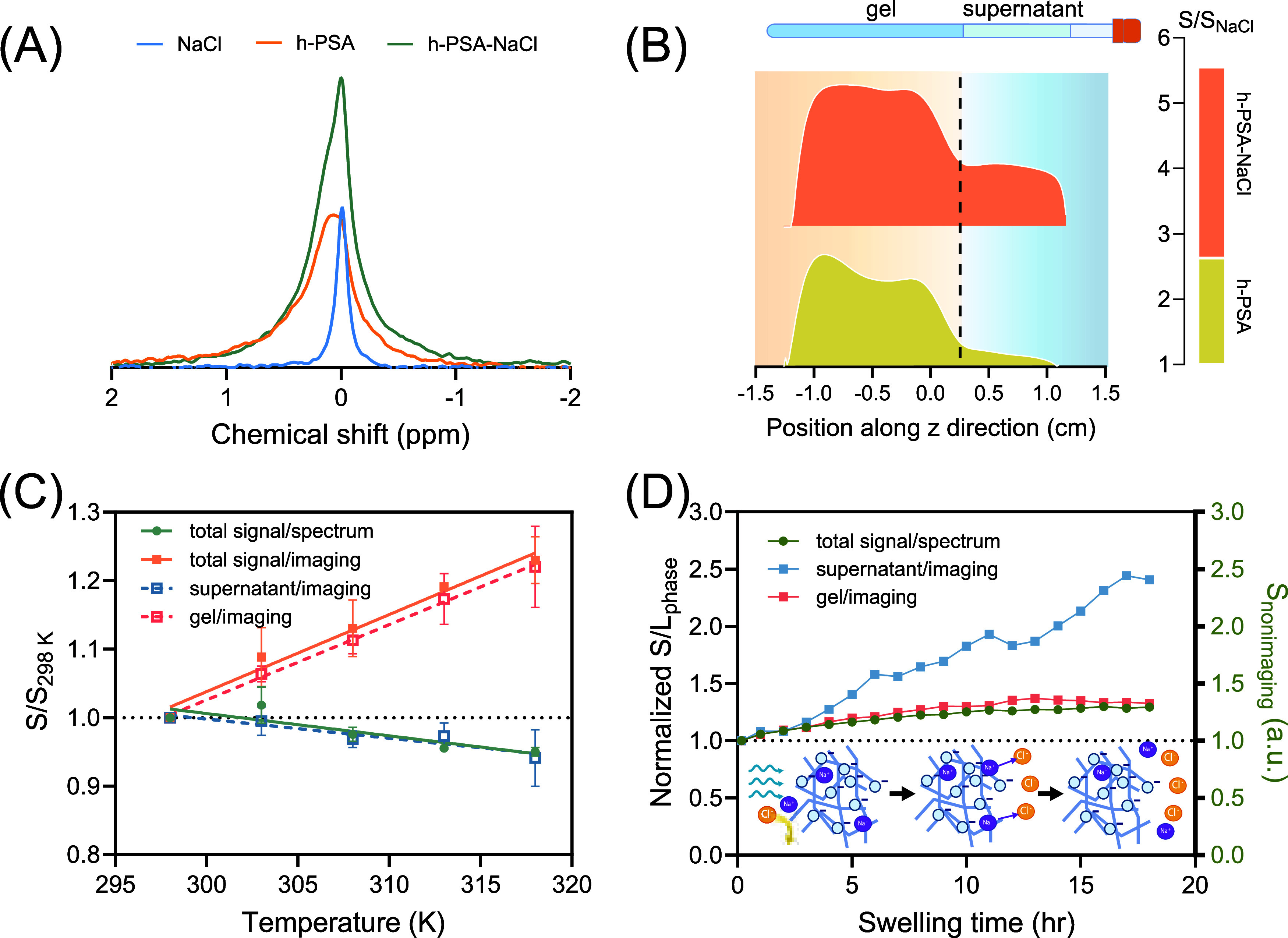
1D ^23^Na NMR spectroscopy and imaging analysis of half-filled PSA hydrogels in 17.1 mM NaCl solution. (A) ^23^Na 1D spectra and (B) 1D imaging profiles of half-PSA hydrogel, half-supernatant (h-PSA) as indicated in the schematic of the NMR tube shown above. One sample was prepared by combining PSA with pure D_2_O (henceforth PSA/D_2_O), and the other by combining it with a 17.1 mM NaCl solution (in D_2_O) at 298 K (henceforth PSA/NaCl). The bar plot indicates the relative signal integral (S/S_NaCl_) of each sample compared to the signal integral of a 17.1 mM NaCl solution. (C) Temperature dependence of the normalized total signal integral of the PSA/NaCl sample measured using spectroscopy (green) vs imaging sequence (orange). The normalized signal integrals of the supernatant phase (blue) and the gel region (red) from 1D imaging profiles are also plotted individually. The error bars were calculated by repeating the measurement two times with h-PSA/NaCl prepared separately. (D) Signal integrals over the length of corresponding phases of the half-filled PSA/NaCl sample vs swelling time. The plot included the total signal (S_nonimaging_) measured from a pulse-acquire spectrum (circle) and the signal over the length of the phase (S/L_phase_) for each of the two phases separately, measured from 1D imaging (square). The S/L_phase_ values are normalized by the signal integral at 0.25 h from the corresponding regions.

In the spectra in Figure 1A, we also observed that the resonance characteristic of ^23^Na in hydrogels was shifted to a higher frequency compared to the peak of the NaCl solution. For the h-PSA-NaCl sample, the two signals overlapped significantly, and their relative contributions were determined via signal deconvolution (see Section III in the Supporting Information).

Moreover, the h-PSA-NaCl sample showed a clear interface between the swollen hydrogel region in the lower half and the salt solution remaining at the top of the NMR tube (supernatant). One-dimensional (1D) imaging along the long axis of the tube can be used to obtain signals from both regions separately. The corresponding regions and extents of each phase are clearly visible in the 1D ^23^Na NMR imaging profiles (Figure 1B). The *z* coordinate position in the 1D ^23^Na NMR imaging profile was calculated based on the formula:^32,33^ $z=\frac{\omega_{G}}{\gamma G_{z}}$ , where ω_*G*_ is the observed frequency shift caused by the gradient, γ is the gyromagnetic ratio of the nucleus (in this case ^23^Na), and *G*_*z*_ is the strength of the acquisition gradient along the *z* direction.

It is known that in liquid-state NMR spectrometers, the pulsed field gradients exhibit some degree of nonlinearity toward the edge, and the radiofrequency field also becomes inhomogeneous and decreases toward zero at larger distances, both of which lead to artifacts in the form of signal humps obtained from the regions at the periphery of the active radiofrequency coil region.^34–36^ To correct for these effects, we divide the 1D imaging profiles by a reference image obtained from a NaCl solution (see Figure S8 in the Supporting Information). Although an additional correction could be made to completely linearize the *z*-axis based on the known gradient nonlinearity, the procedure used here was sufficient for our purposes of separating the two regions.^36^ To avoid unrealistic noise amplification at the edges of the detection volume (due to division by a small reference signal), the normalization procedure was only applied within the *z* region from −1 to 1 cm. The edge regions of the imaging profile beyond this range were left unnormalized and were scaled to produce a smooth continuation of the normalized signal in the regions |*z*|> 1 cm. All subsequent quantitative analyses of the 1D imaging profiles were performed only on the normalized regions to avoid misquantification due to abnormalities in the edge region.

The position of the gel-supernatant interface was estimated to be located at approximately *z* ∼ 0.2 cm, where the 1D images show a sharp discontinuity. The signal from the supernatant phase was integrated from ∼0.2 to 1 cm in the spectrum, while the gel region signal was integrated from *–*1 to 0.2 cm. In the imaging profile of the h-PSA/D_2_O sample, the supernatant phase signal arises from the small portion of Na^+^ ions seeping out from the hydrogels. We immediately notice that very little Na^+^ goes into the supernatant phase, indicating that the sodium originally contained in the PSA is relatively strongly interacting with the matrix there.

The h-PSA/NaCl sample showed a much stronger ^23^Na signal in the supernatant phase. From these two extreme situations, it becomes clear that this type of imaging analysis can be very useful in determining the relative concentrations of sodium ions in the different environments and for performing Donnan equilibrium calculations, potentially in situ during swelling or compression processes.

Figure 1C demonstrates that we may use the signal integrals obtained from the hydrogel and supernatant regions for quantification of the different sodium pools. The signal integrals from the two regions correspond reasonably well to the signal integrals obtained by ^23^Na spectroscopy from full tubes of either the hydrogel or just saline solution over the examined temperature range, as shown here. This method of signal analysis, separating the gel region and supernatant phase, allows for a further investigation of ^23^Na ion migration (reflecting bulk dynamics) and nuclear polarization lifetime measurements (reflecting microscopic dynamics) during the hydrogel desalination process, as discussed further below. The signals are all normalized to the signal observed at the lowest temperature for the ease of comparison of the trends.

The ^23^Na signal increased toward higher temperatures in the hydrogel region, while it decreased in the supernatant region. The signal decrease of the saline and supernatant regions with increasing temperatures can be well explained by the change in the Boltzmann distribution, where nuclear magnetization is inversely proportional to absolute temperature.^37^

By contrast, the increasing signals in the gel region toward higher temperatures can be explained by the speedup of ion motion. Consequently, transverse polarization lifetimes increase, and more of the signal survives the echo time used in the imaging experiment. Some strongly bound sodium remains significantly attenuated due to the finite echo time needed for the imaging experiment (several milliseconds), during which signals from those ions decay. The bound sodium fraction is analyzed in more detail below.

In addition to the changes in tumbling rates, the temperature effect on the overall hydrogel matrix could also be a significant factor in the observed effect. The cross-linked matrix becomes less stable as the temperature increases, which causes more salt to enter the hydrogel phase, rendering it less efficient for ion exclusion, with more weakly bound sodium residing in the gel region.^27,38,39^ This decrease in salt rejection efficiency could also be an additional reason for the decrease of the ^23^Na signal with increasing temperature from the supernatant phase shown in the 1D imaging profiles.

Sequential 1D ^23^Na NMR imaging experiments were performed to track the migration of Na^+^ ions during the hydrogel swelling process. The spectra were continuously acquired for swelling times from 0.25 to 18 h at room temperature (Figure 1D). The supernatant phase showed a clear increase as time progressed. The signal from the gel region also increased with time, but to a smaller degree. Because the dried PSA hydrogels used in our measurements are numerous small clusters instead of one single matrix, the gel region contains not only the swollen hydrogels but also the NaCl solution between those clusters. Although the ^23^Na signals from the gel region were majorly contributed by the swollen hydrogels, the surrounding NaCl solution also contributed to the signals and could be another reason for the slight signal increment in the gel region as the swelling process proceeded, in addition to the extension of hydrogel volume. The ionic imbalance across the interface drives the free Na^+^ ions to the surrounding solution to balance with the excluded Cl^–^ ions; therefore, the supernatant signal increases rapidly with time. The slower increase in the signal from the gel–solution phase can be explained by the expansion of the hydrogel volume before reaching the swelling equilibrium^38^ which in turn increases the population of the freely tumbling sodium. This statement was further proved by measuring the R_2_ values as a function of swelling time (see Figure S7 in the Supporting Information).

### 3.2 Relaxation Behavior under Different Salt Conditions

Seawater represents an electrolyte mixture of various cations and anions in high concentrations, leading to complex ion and water dynamics.^3^ Thus, it is important to consider the effect of competing salts on the desalination performance of the hydrogels. We explore the behavior of several salt environments by adding salts with competing cation effects to the NaCl solution, such as KCl or MgCl_2_, and measure ^23^Na relaxation rates in each case to analyze the binding dynamics between Na^+^ ions and hydrogels in these systems. The focus of competing cation effects for this study is motivated by the anionic nature of the PSA hydrogel with fixed carboxylate groups, which directly interact with cations through charge compensation and electrostatic effects. K^+^ and Mg^2+^ ions were selected specifically because these ions (together with Na^+^) represent major cations that are naturally present in seawater.^3^ Furthermore, it is of interest to examine the effect of multivalent ions.

Two situations were investigated: competing salts were added, and the NaCl was reduced (i) to give the same total concentration and (ii) to give the same total ionic strength as the 17.1 mM NaCl solution. The purpose of preparing these two control groups is to separate the contributions of the total ionic strength from ion-specific interactions to the changes in ^23^Na relaxation rates. Different cations alter the dynamic structure of hydrogen bonds in bulk water and/or compete for binding or association at the negatively charged carboxylate groups in the hydrogel.^40^ If we only control for concentration, the major effect on the relaxation rates of Na^+^ ions would arise from the differences in the ionic strength and Debye length.^41,42^

No clear differences were observed in the 1D ^23^Na imaging profiles of half-filled PSA hydrogels prepared with a NaCl+KCl solution and a NaCl+MgCl_2_ solution with the same total ionic strength, while a higher supernatant signal was observed for concentration-normalized samples (Figure 2A). This finding indicates that more free Na^+^ ions have been released from the gel phase due to an unbalanced ionic potential. Moreover, the larger signal peak of the supernatant phase for the sample in the NaCl+MgCl_2_ solution can be explained by the doubly charged Mg^2+^ ions strongly binding to the negatively charged polymer chains, which results in more Na^+^ ions floating out from the hydrogel phase.^43,44^

**Figure 2:**
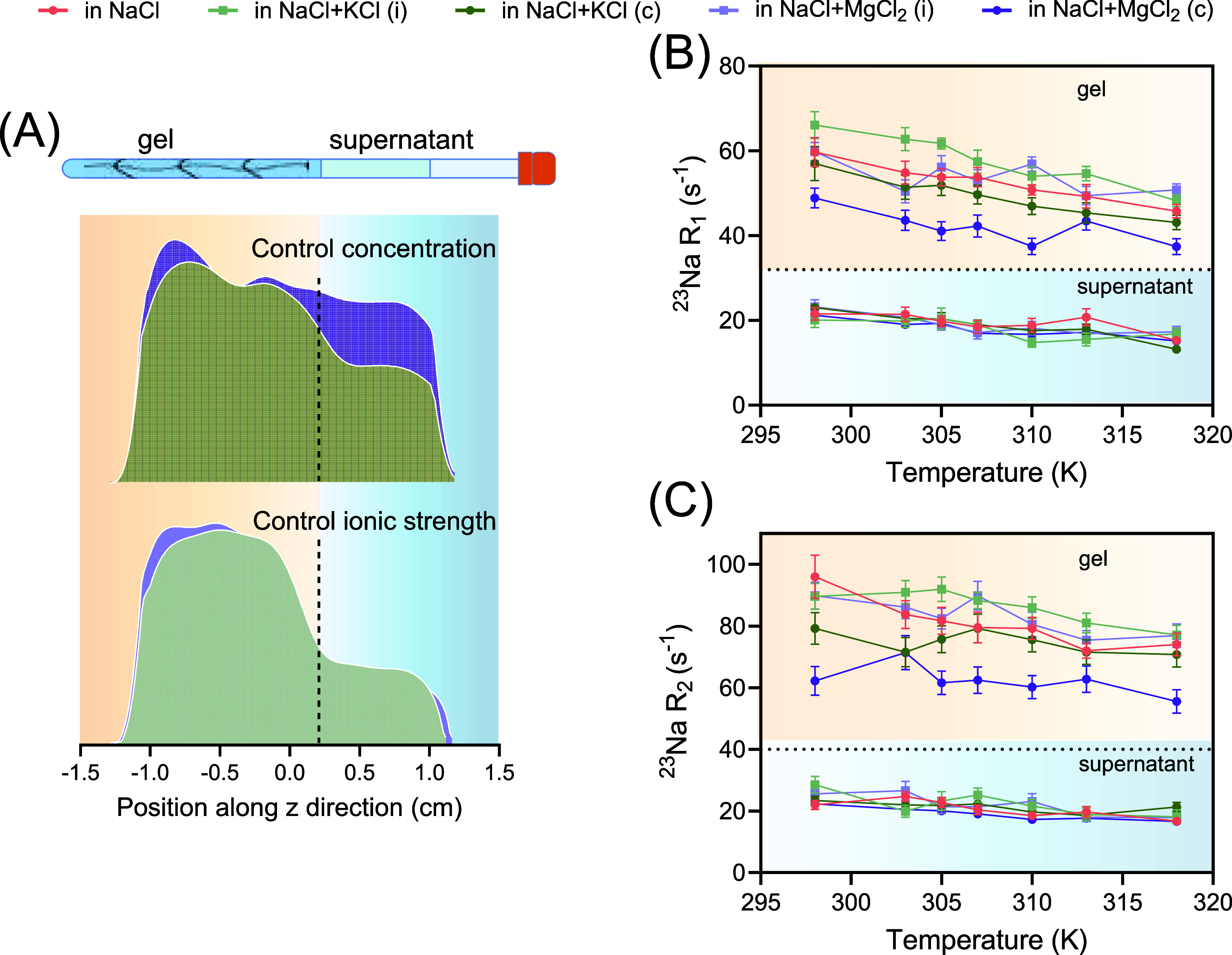
^23^Na *R*_1_ and *R*_2_ relaxation rates from PSA/supernatant samples prepared with different salt solutions using relaxation-edited imaging experiments. (A) 1D ^23^Na imaging profiles of half-filled PSA in NaCl+KCl (green) and NaCl+MgCl_2_ (purple) solutions under concentration control (top, c) and ionic strength control (bottom, i) conditions. (B) The ^23^Na longitudinal relaxation rate (*R*_1_) and (C) transverse relaxation rate (*R*_2_) of the gel region and supernatant phase for samples in NaCl (red), NaCl+KCl, and NaCl+MgCl_2_ solutions with controlled concentration (circle, dark) and controlled ionic strength (square, light) over the temperature range from 298 to 318 K.

Next, we measured the ^23^Na relaxation rate constants using *T*_1_-edited imaging (T1SR_img_) and *T*_2_-edited imaging (CPMG_img_) pulse sequences (see Figure S1 in the Supporting Information). The signal contributions of the supernatant phase and the gel region were integrated separately, which allowed measuring the spatially resolved relaxation rates *R*_1_ = 1/*T*_1_ and *R*_2_ = 1/*T*_2_ of the free Na^+^ ions in the supernatant, and estimating the relaxation rates corresponding to the slow-tumbling Na^+^ ions within the gel region. As anticipated, the relaxation rates decreased with increasing temperature in the gel region, while the decreasing trend was less pronounced in the supernatant phase (Figure 2B,C). This decrease was observed for all salt solutions. Small variations are shown for both *R*_1_ and *R*_2_ of the supernatant phase as values remain nearly constant at around 20 s^–1^. For samples in multisalt solutions with matched ionic strength, the gel region showed that *R*_1_ and *R*_2_ values were close to the ones for h-PSA/NaCl, while the ones with matched concentration showed smaller relaxation rates, again indicative of a larger fraction of free sodium becoming available.

Smaller ^23^Na *R*_1_ and *R*_2_ values in the gel region were measured for NaCl+MgCl_2_ samples than for NaCl+KCl samples. This difference results from the stronger electrostatic interactions between Mg^2+^ ions and fixed charge groups, and some Na^+^ ions brought from the PSA hydrogel itself were expelled into the surrounding solution. Although the amount of Mg^2+^ ions in seawater is much lower than that of Na^+^ ions,^3^ it is important to note that the effect of Mg^2+^ ions on the desalination performance of PSA hydrogels is significant and should be considered for assessing desalination performance.

Next, we investigated possible chemical exchange phenomena of Na^+^ ions between different sites that might increase the apparent transverse relaxation rate. No relaxation rate dispersion was observed when increasing the CPMG interecho spacing from 2 to 300 ms, implying that no significant exchange dynamics takes place on this time scale (see Section IV in the Supporting Information).

### 3.3 Quadrupolar Couplings Probed by TQF NMR

The hydrogel region is likely not a completely uniform phase but consists of regions of variability in density and water content. It is reasonable to assume that the gel region also contains a large amount of free sodium ions. We investigated this question further in the following spectroscopy and imaging experiments within the hydrogel region (Figures 3 and 4).

**Figure 3:**
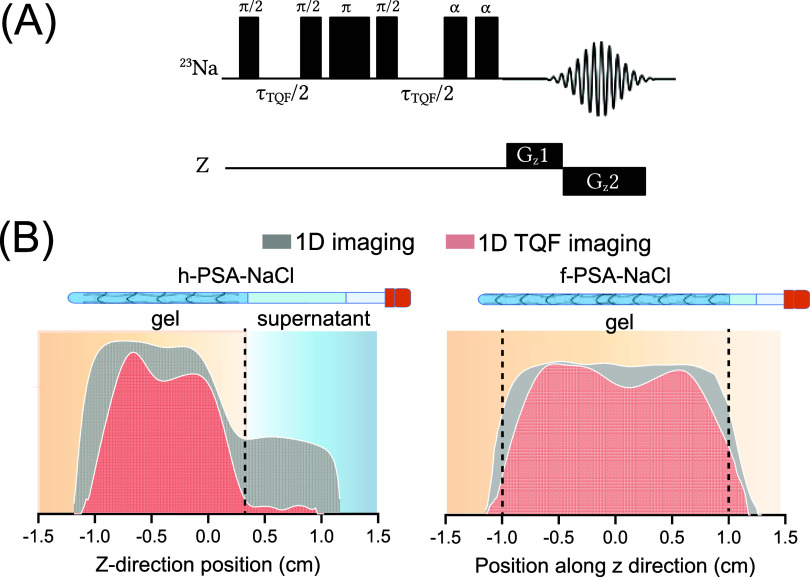
^23^Na TQF imaging experiments of half-filled and full-tube PSA in 17.1 mM NaCl solution. (A) The 1D TQF imaging pulse sequences with α = 90°. (B) The 1D TQF imaging profile of h-PSA/NaCl (left) and f-PSA/NaCl (right) obtained at the maximum signal position. The delay τ_TQF_ at which the signal is maximum for the h-PSA/NaCl solution is 8 ms, and for f-PSA/NaCl it is 6 ms. The 1D single-pulse imaging profile and the 1D TQF imaging profile of the sample are shown in gray and red colors, respectively.

**Figure 4:**
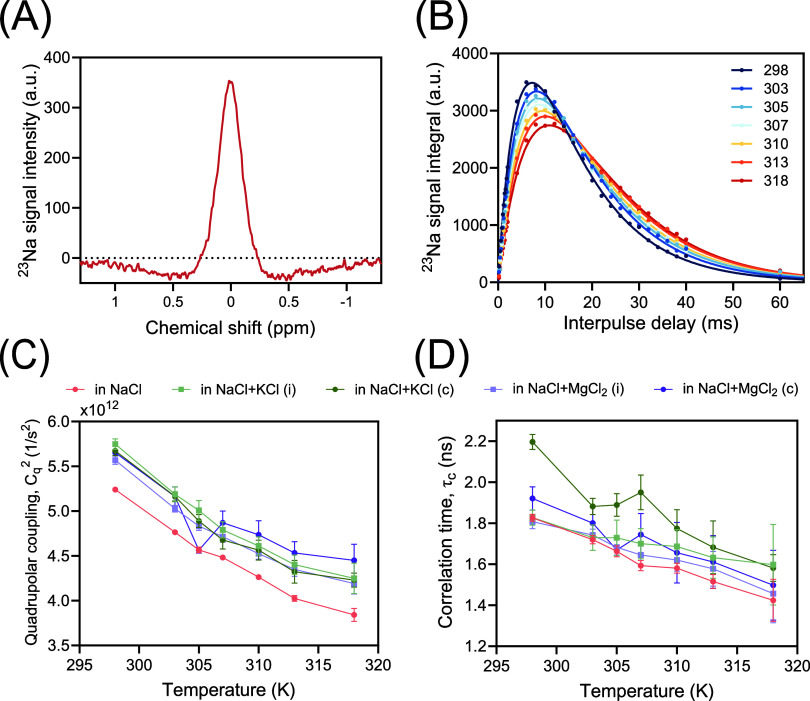
^23^Na TQF experiments of full-tube PSA prepared with different salt solutions. (A) The 1D nonimaging TQF spectrum (red) of full-tube PSA in NaCl solution (f-PSA/NaCl) at room temperature and τ_TQF_ = 6 ms compared with a 1D pulse-acquired spectrum (black). (B) TQF signal integral as a function of τ_TQF_ of full-tube PSA/NaCl for different temperatures. (C) The quadrupolar coupling constant and (D) the correlation time extracted from the TQF buildup curves of multisalt solutions (as indicated by the legend) vs temperature.

To selectively measure the signal from Na^+^ ions in the hydrogel, triple-quantum filter (TQF) NMR experiments (Figure 3A) were performed to isolate the contribution of Na^+^ ions that experience slowly modulated quadrupolar interactions.^45–47^

We implemented the TQF experiment for selective imaging of the half-filled PSA and full-tube PSA samples, where the hydrogel phase covers either half or all of the detection region (Figure 3). TQF-edited imaging experiments have previously been used to study soft biological tissues,^48–50^ and here we extend the use of TQF imaging to the PSA hydrogel desalination system, to investigate the distribution of bound sodium over the volume. The 1D TQF imaging profile of the half-filled PSA and full-tube PSA in NaCl solution was measured at τ_TQF_ = 8 and 6 ms, respectively, corresponding to the highest signal intensity derived from spectroscopy experiments (Figure 4A). Compared to the single-pulse imaging profile of the half-tube PSA in NaCl solution, no signal was shown from the supernatant phase (0.2–1 cm region) in the 1D TQF imaging profile, while clear signals were shown from the gel region (Figure 3B, left), thereby also confirming the filtering quality of the TQF sequence. For the full-tube PSA in NaCl solution, the whole detection region showed clear signals (Figure 3B, right).

In Figure 4A, the 1D TQF spectrum of the full-tube PSA in NaCl solution is shown for the interpulse delay that maximizes the signal intensity. The baseline distortion observed in the TQF ^23^Na spectrum results from the superposition of peaks with different relaxation rates at the same frequency.^30,51^ In the pulse-acquire 1D spectrum, the ^23^Na resonance appeared as an asymmetric peak because signals from free, loosely bound, and strongly bound Na^+^ ions all overlapped into one peak near 0 ppm. By contrast, the TQF spectrum showed a characteristic symmetric peak as the TQF selectively suppressed the rapidly tumbling free ions and showed only signals arising from slow-tumbling Na^+^ ions. This finding is very clear evidence of the ability of ^23^Na spectroscopy to measure specifically the strongly bound sodium ions in the hydrogel phase.

The ^23^Na TQF spectrum integral measured as a function of mixing time τ_TQF_ is well described by the biexponential function characteristic of the presence of bound or slowly tumbling sodium ions,^45–47^ *S*(τ_TQF_) = *A*e^–*R*_2s_ τ_TQF_^ – *A*e^–*R*_2f_ τ_TQF_^, where *R*_2s_ and *R*_2f_ represent the slow and fast transverse relaxation rates giving rise to the build-up profile. In terms of spectral density components *J*(ω), these rates are expressed as^52^ *R*_2f_ = 3*J*(0) + 3*J*(ω_0_) and *R*_2s_ = 3*J*(ω_0_) + 3*J*(2ω_0_). The spectral density function for quadrupolar nuclei is $J\left(\omega \right)=\frac{2}{5}C_{Q}^{2}\frac{\tau_{c}}{1+{\left(\omega \tau_{c}\right)}^{2}}$ , where ω is the Larmor frequency of the ^23^Na nucleus, τ_*c*_ is the correlation time describing the modulation of the ^23^Na interaction, and *C*_*Q*_ is the quadrupolar coupling constant given by $C_{Q}^{2}=\frac{e^{2}Q^{2}\left\langle V^{2}\right\rangle }{36\hbar^{2}}$ with *e* as the elementary charge, ℏ the reduced Planck constant, *Q* the quadrupole moment of the ^23^Na nucleus, and ⟨ ***V*** ^**2**^⟩ the variance of the fluctuating electric field tensor.^29^ The quadrupolar coupling is a measure of the asymmetry of the solvation shell, and the correlation time is a measure of the dynamics of the environment.

By fitting the TQF profiles (Figure 4B) at different temperatures, we observed a decrease in both the quadrupolar coupling constant *C*_*Q*_ and the correlation time τ_*C*_ with increasing temperatures for all samples (Figure 4C,D). A reduction in the average quadrupolar interaction means that the binding effect weakens, and the reduction in correlation time means that dynamics becomes faster with increasing temperatures, both effects that seem intuitive for the systems under study. Such findings could be used to specifically investigate the extent of salt rejection functions of different hydrogel media.

Because K^+^ ions have a weaker affinity for the carboxylate groups than Na^+^ ions, they tend to remain in the supernatant and contribute to the total ionic strength.^53^ As shown in Figure 4D, the Na^+^ ions within the gel region in NaCl+KCl solution showed a higher correlation than in NaCl or in NaCl+MgCl_2_ solution under both controlled concentration time and controlled total ionic strength conditions. One possible explanation is that some of the free Na^+^ ions in the supernatant were replaced by K^+^ ions, forcing more Na^+^ ions to be associated with the hydrogel. For PSA in NaCl+MgCl_2_ solution, the presence of Mg^2+^ ions leads to a more restricted hydrogel matrix, thereby also slowing the motion of the surrounding Na^+^ ions compared to samples in NaCl solution. However, as indicated in the relaxation rate analysis (Figure 2B,C), the relaxation rates within the gel region in NaCl+MgCl_2_ showed significantly smaller values at a controlled concentration, which also indicated that Mg^2+^ ions displaced a portion of Na^+^ ions from the hydrogels into the supernatant, thus reducing the overall contribution of strongly bound Na^+^ ions.

## 4 Conclusions

This work addresses the study of ion dynamics in the polyelectrolyte hydrogel desalination process. While these hydrogels are promising solutions for fresh water supplies, optimizing them has been difficult because standard testing methods (such as measuring ionic conductivity) only focus on overall salinity changes, but do not inform on how or where the ions are moving inside the material, or how structure influences the different salt pools. To overcome this limitation, we implemented ^23^Na NMR spectroscopy and imaging techniques, which allowed us to map the sodium distribution across compartments and investigate how the distributions change over time based on parameters such as temperature or the presence of competing ions. Specifically, we also identify a strongly bound sodium pool with the use of TQF NMR. This technique distinguishes between sodium ions that are just passing through (free) and those that are chemically stuck to the hydrogel’s polymer chains (bound). It was also found that raising the temperature sped up ion motion, and the bound pool was characterized by higher mobility and higher symmetry (smaller quadrupolar coupling), likely due to exchange with the free pool. Seawater also contains other ions, and hence we also studied here the influence of competing ions such as potassium and magnesium on the sodium distribution. Specifically, the divalent magnesium ion is shown to have a drastic effect in replacing sodium in the bound pool. This work establishes a new diagnostic tool for hydrogel-based water purification materials. By using the protocols developed here (relaxation and TQF imaging), one can examine desalination happening in real-time. This approach paves the way for designing “smart” hydrogels that are tuned to reject specific salts more efficiently, potentially lowering the energy cost and increasing the efficiency of producing fresh water.

## Supplementary Material



## Notes

Additional experimental methods and results; TQF NMR setup; deconvolution analysis of ^23^Na 1D NMR spectra; analysis of ionic exchange dynamics; and 1D NMR imaging profiles (PDF)

## Abbreviations

NMR: nuclear magnetic resonance
PSA: sodium polyacrylate
*R*_1_: longitudinal relaxation rate
*R*_2_: transverse relaxation rate
MQF: multiple-quantum-filtered
TQF: triple-quantum filtered

## References

[1] Liu J., Yang H., Gosling S. N., Kummu M., Flörke M., Pfister S., Hanasaki N., Wada Y., Zhang X., Zheng C., Alcamo J., Oki T. (2017). Water Scarcity Assessments
in the Past, Present, and Future. Earth’s
Future.

[2] Salehi M. (2022). Global Water
Shortage and Potable Water Safety; Today’s Concern and Tomorrow’s
Crisis. Environ. Int..

[3] Lyman, J. ; Fleming, R. Composition of Sea Water J. Mar. Res. 1940; Vol. 3.

[4] Werber J. R., Osuji C. O., Elimelech M. (2016). Materials
for Next-Generation Desalination
and Water Purification Membranes. Nat. Rev.
Mater..

[5] Jangizehi A., Seiffert S. (2021). Salt Partitioning in
Ionized, Thermo-Responsive Hydrogels:
Perspective to Water Desalination. J. Chem.
Phys..

[6] Wang Q., Yang Y., Liang S., Wu T., Zhang J., Ji Y., Su Z., Wang C., Geng Z., Huo M. (2025). Solar-Driven
Bifunctional Hydrogel Enables All-Weather Pure Water and Draw Agent
Regeneration for Forward Osmosis. Sep. Purif.
Technol..

[7] Chen G. (2022). Thermodynamics
of Hydrogels for Applications in Atmospheric Water Harvesting, Evaporation,
and Desalination. Phys. Chem. Chem. Phys..

[8] Arens L., Albrecht J. B., Höpfner J., Schlag K., Habicht A., Seiffert S., Wilhelm M. (2017). Energy Consumption
for the Desalination
of Salt Water Using Polyelectrolyte Hydrogels as the Separation Agent. Macromol. Chem. Phys..

[9] Salehi A. A., Ghannadi-Maragheh M., Torab-Mostaedi M., Torkaman R., Asadollahzadeh M. (2021). Hydrogel Materials
as an Emerging Platform for Desalination and the Production of Purified
Water. Sep. Purif. Rev..

[10] Hua F., Qian M. (2001). Synthesis of Self-Crosslinking
Sodium Polyacrylate Hydrogel and Water-Absorbing
Mechanism. J. Mater. Sci..

[11] Arens L., Barther D., Landsgesell J., Holm C., Wilhelm M. (2019). Poly­(Sodium
Acrylate) Hydrogels: Synthesis of Various Network Architectures, Local
Molecular Dynamics, Salt Partitioning, Desalination and Simulation. Soft Matter.

[12] Höpfner J., Klein C., Wilhelm M. (2010). A Novel Approach for the Desalination
of Seawater by Means of Reusable Poly­(Acrylic Acid) Hydrogels and
Mechanical Force. Macromol. Rapid Commun..

[13] Meka V. S., Sing M. K. G., Pichika M. R., Nali S. R., Kolapalli V. R. M., Kesharwani P. (2017). A Comprehensive
Review on Polyelectrolyte Complexes. Drug Discovery
Today.

[14] Yu Y., Peng R., Yang C., Tang Y. (2015). Eco-Friendly and Cost-Effective
Superabsorbent Sodium Polyacrylate Composites for Environmental Remediation. J. Mater. Sci..

[15] Ali W., Gebert B., Hennecke T., Graf K., Ulbricht M., Gutmann J. S. (2015). Design of Thermally Responsive Polymeric Hydrogels
for Brackish Water Desalination: Effect of Architecture on Swelling,
Deswelling, and Salt Rejection. ACS Appl. Mater.
Interfaces.

[16] Goh P. S., Ismail A. F., Ng B. C. (2013). Carbon Nanotubes for Desalination:
Performance Evaluation and Current Hurdles. Desalination.

[17] Gilron J., Gara N., Kedem O. (2001). Experimental Analysis
of Negative
Salt Rejection in Nanofiltration Membranes. J. Membr. Sci..

[18] Mathur A. M., Scranton A. B. (1996). Characterization of Hydrogels Using
Nuclear Magnetic
Resonance Spectroscopy. Biomaterials.

[19] Li P., Malveau C., Zhu X. X., Wuest J. D. (2022). Using Nuclear Magnetic
Resonance Spectroscopy to Probe Hydrogels Formed by Sodium Deoxycholate. Langmuir.

[20] Demco D. E., Pich A. (2023). Structure and Dynamics of Temperature-Responsive
Microgels and Hydrogels
by NMR Spectroscopy, Relaxometry, and Diffusometry. Macromol. Chem. Phys..

[21] Guo X., Theissen S., Claussen J., Hildebrand V., Kamphus J., Wilhelm M., Luy B., Guthausen G. (2019). Dynamics of
Sodium Ions and Water in Swollen Superabsorbent Hydrogels as Studied
by 23Na- and 1H-NMR. Macromol. Chem. Phys..

[22] Krakovský I., Hanyková L., Štastná J. (2025). Phase Transition
in
Polymer Hydrogels Investigated by Swelling, DSC, FTIR and NMR. J. Therm. Anal. Calorim..

[23] Wang R., Xin J., Ji Z., Zhu M., Yu Y., Xu M. (2023). Spin-Space-Encoding
Magnetic Resonance Imaging: A New Application for Rapid and Sensitive
Monitoring of Dynamic Swelling of Confined Hydrogels. Molecules.

[24] Arndt, K.-F. ; Knörgen, M. ; Richter, S. ; Schmidt, T. Modern Magnetic Resonance; Webb, G. A. , Ed.; Springer Netherlands: Dordrecht, 2006; pp 187–193.

[25] Ozel B., Uguz S. S., Kilercioglu M., Grunin L., Oztop M. H. (2017). Effect
of Different Polysaccharides on Swelling of Composite Whey Protein
Hydrogels: A Low Field (LF) NMR Relaxometry Study. J. Food Process Eng..

[26] Keeler, J. Understanding NMR Spectroscopy, 2nd ed.; John Wiley and Sons: Chichester, U.K., 2010.

[27] Kipcak A. S., Ismail O., Doymaz I., Piskin S. (2014). Modeling and Investigation
of the Swelling Kinetics of Acrylamide-Sodium Acrylate Hydrogel. J. Chem..

[28] Kowalewski, J. ; Maler, L. Nuclear Spin Relaxation in Liquids: Theory, Experiments, and Applications; CRC Press: Boca Raton, 2006.

[29] Chubak I., Alon L., Silletta E. V., Madelin G., Jerschow A., Rotenberg B. (2023). Quadrupolar
23Na + NMR Relaxation as a Probe of Subpicosecond
Collective Dynamics in Aqueous Electrolyte Solutions. Nat. Commun..

[30] Kemp-Harper R., Brown S. P., Hughes C. E., Styles P., Wimperis S. (1997). Erratum to
“23Na NMR Methods for Selective Observation of Sodium Ions
in Ordered Environments”. Prog. Nucl.
Magn. Reson. Spectrosc..

[31] Salman, S. R. Encyclopedia of Spectroscopy and Spectrometry, Second ed.; Lindon, J. C. , Ed.; Academic Press: Oxford, 1999; pp 246–252.

[32] McRobbie, D. W. ; Moore, E. A. ; Graves, M. J. ; Prince, M. R. MRI From Picture to Proton, Second ed.; Cambridge University Press: Cambridge, United Kingdom, 2007; pp 65–71.

[33] Hornak, J. P. Basics of MRI. In Interactive Learning Software, Chapter 6; Henrietta, NY, 2012 http://www.cis.rit.edu/htbooks/mri/.

[34] Jerschow A., Bodenhausen G. (1999). Mapping theB1Field
Distribution with Nonideal Gradients
in a High-Resolution NMR Spectrometer. J. Magn.
Reson..

[35] Hoult D. I., Richards R. E. (1976). The Signal-to-Noise Ratio of the Nuclear Magnetic Resonance
Experiment. J. Magn. Reson..

[36] Yarach U., Luengviriya C., Danishad K., Stucht D., Godenschweger F., Schulze P., Speck O. (2015). Correction of Gradient Nonlinearity
Artifacts in Prospective Motion Correction for 7T MRI. Magn. Reson. Med..

[37] Abragam, A. Principles of Nuclear Magnetism, International Series of Monographs on Physics; Oxford University Press: Oxford, NY, 1983.

[38] Mahon R., Balogun Y., Oluyemi G., Njuguna J. (2020). Swelling Performance
of Sodium Polyacrylate and Poly­(Acrylamide-Co-Acrylic Acid) Potassium
Salt. SN Appl. Sci..

[39] Wang Y., He G., Li Z., Hua J., Wu M., Gong J., Zhang J., Ban L.-t., Huang L. (2018). Novel Biological Hydrogel:
Swelling Behaviors Study in Salt Solutions with Different Ionic Valence
Number. Polymers.

[40] Zhang C., Jerschow A. (2024). Range and Sensitivity of 17O Nuclear Spin-Lattice Relaxation
as a Probe of Aqueous Electrolyte Dynamics. J. Chem. Phys..

[41] Feng Y., Taraban M., Yu Y. B. (2012). The Effect
of Ionic Strength on the
Mechanical, Structural and Transport Properties of Peptide Hydrogels. Soft Matter.

[42] Dong Y., Laaksonen A., Gao Q., Ji X. (2021). Molecular Mechanistic
Insights into the Ionic-Strength-Controlled Interfacial Behavior of
Proteins on a TiO2 Surface. Langmuir.

[43] Pinzon-Moreno D. D., Maurate-Fernandez I. R., Flores-Valdeon Y., Neciosup-Puican A. A., Carranza-Oropeza M. V. (2022). Degradation
of Hydrogels Based on Potassium and Sodium
Polyacrylate by Ionic Interaction and Its Influence on Water. Polymers.

[44] Kadhim S. A., Hameed A. M., Rasheed R. T. (2022). Synthesis and Study of Magnesium
Complexes Derived from Polyacrylate and Polyvinyl Alcohol and Their
Applications as Superabsorbent Polymers. J.
Mech. Behav. Mater..

[45] Fonseca C. P., Fonseca L. L., Montezinho L. P., Alves P. M., Santos H., Castro M. M. C. A., Geraldes C. F. G. C. (2013). 23Na Multiple Quantum Filtered NMR
Characterisation of Na+ Binding and Dynamics in Animal Cells: A Comparative
Study and Effect of Na+/Li+ Competition. Eur.
Biophys. J..

[46] Eliav U., Shinar H., Navon G. (1992). The Formation of a Second-Rank Tensor
in 23Na Double-Quantum-Filtered NMR as an Indicator for Order in a
Biological Tissue. J. Magn. Reson..

[47] Eykyn T. R., Aksentijević D., Aughton K. L., Southworth R., Fuller W., Shattock M. J. (2015). Multiple
Quantum Filtered (23)­Na
NMR in the Langendorff Perfused Mouse Heart: Ratio of Triple/Double
Quantum Filtered Signals Correlates with [Na]­i. J. Mol. Cell. Cardiol..

[48] Ooms K. J., Cannella M., Vega A. J., Marcolongo M., Polenova T. (2008). 23Na TQF NMR Imaging for the Study of Spinal Disc Tissue. J. Magn. Reson..

[49] Tsoref L., Shinar H., Navon G. (1998). Observation of a 1H
Double Quantum
Filtered Signal of Water in Biological Tissues. Magn. Reson. Med..

[50] Tsang A., Stobbe R. W., Beaulieu C. (2012). Triple-Quantum-Filtered Sodium Imaging
of the Human Brain at 4.7 T. Magn. Reson. Med..

[51] Jaccard G., Wimperis S., Bodenhausen G. (1986). Multiple-quantum
NMR Spectroscopy
of S = 3/2 Spins in Isotropic Phase: A New Probe for Multiexponential
Relaxation. J. Chem. Phys..

[52] Madelin G., Lee J.-S., Regatte R. R., Jerschow A. (2014). Sodium MRI: Methods
and Applications. Prog. Nucl. Magn. Reson. Spectrosc..

[53] Cheng Y., Korolev N., Nordenskiöld L. (2006). Similarities
and Differences in Interaction
of K+ and Na+ with Condensed Ordered DNA. A Molecular Dynamics Computer
Simulation Study. Nucleic Acids Res..

