# Supplementary material for: Revealing Hidden Dynamics of Hydrogel-Based Desalination with 23Na Nuclear Magnetic Resonance
Source: J Phys Chem C Nanomater Interfaces. 2026 Jun 4;130(24):8390–8. doi: 10.1021/acs.jpcc.6c02171 (PMC13288675; doi:10.1021/acs.jpcc.6c02171)
Supplement: Supplementary file 1 [file jp6c02171_si_001.pdf]

Supporting Information: Revealing Hidden Dynamics of Hydrogel-based Desalination with ^{23}Na Nuclear Magnetic Resonance

Huijing Zou,† Chengtong Zhang,† Florin Teleanu,†,‡ Amir Jangizehi,¶ Sebastian Seiffert,¶ and Alexej Jerschow*†

†*Department of Chemistry, New York University, 100 Washington Square East, New York, 10003, NY, United States*

‡*ELI-NP, "Horia Hulubei" National Institute for Physics and Nuclear Engineering, 30 Reactorului Street, Bucharest-Magurele, 077125, Ilfov, Romania*

¶*Department of Chemistry, Johannes Gutenberg-Universität Mainz, Duesbergweg 10–14, D-55128 Mainz, Rhineland Palatinate, Germany*

This Supporting Information contains additional NMR measurement protocols, experimental data, pulse sequences and description of data-analysis workflow referenced in the main text. The document is organized as follows:

- Section I: T_1, T_2-edited imaging pulse sequences.
- Section II: Experimental methods
- Section III: Deconvolution of ^{23}Na 1D zg NMR spectra.
- Section IV: Investigating potential ionic exchange dynamics.

- Section V: 1D imaging profiles.

Section I: T_1, T_2 edited imaging pulse sequences

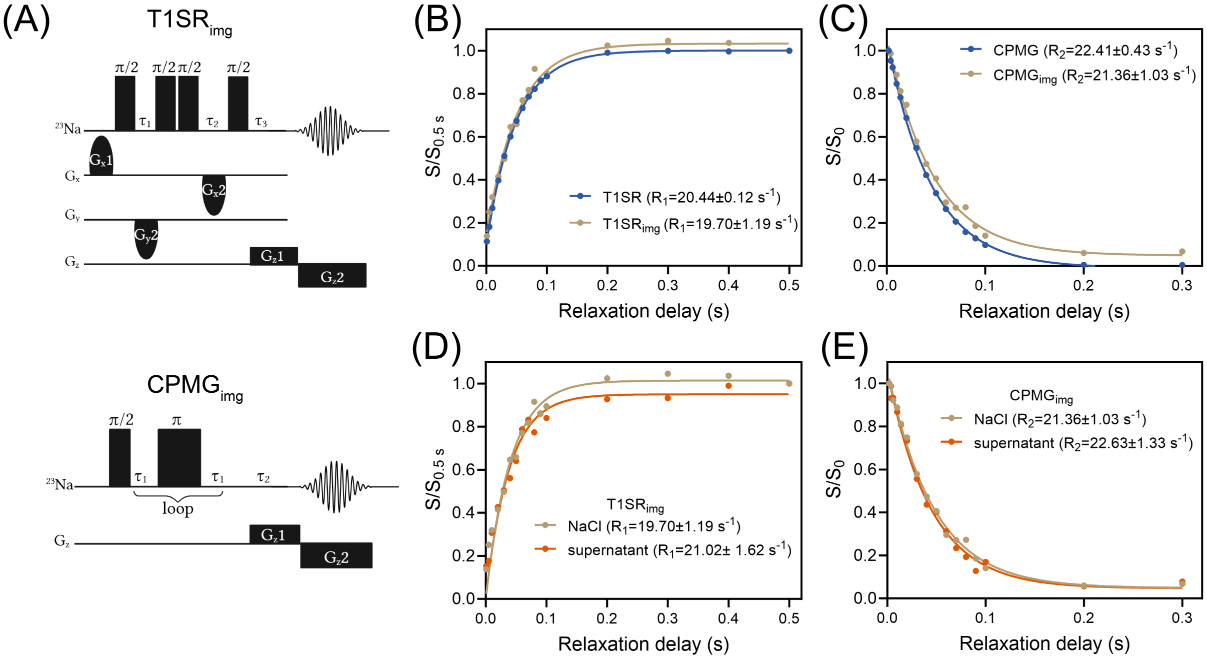

Figure S1. The ^{23}Na relaxation rates of NaCl solution and the supernatant of half-filled PSA in NaCl solution at room temperature using both imaging and nonimaging measurements.(A) The T1SR edited ($T1SR_{img}$) and CPMG edited ($CPMG_{img}$) imaging pulse sequences. (B) The ^{23}Na R_1 comparison of NaCl solution between imaging and standard T1SR sequence from normalized signal fitting results. (C) The ^{23}Na R_2 comparison of NaCl solution between imaging and standard CPMG sequence. (D) The ^{23}Na imaging R_1 results for NaCl solution and supernatant. (E) The ^{23}Na imaging R_2 results for NaCl solution and supernatant.

Section II: TQF NMR Measurements

In the triple-quantum-filtered (TQF) NMR experiments, the 90° pulse was applied with phase cycling ph1 = {30°, 90°, 150°, 210°, 270°, 330°}, while the 180° pulse was applied with ph2={120°, 180°, 240°, 300°, 0°, 60°}, where each phase increment is repeated twice with a total of 12 steps. The standard TQF NMR pulse sequence was also modified by adding a gradient echo at the end for imaging purposes. The interpulse delay values (τ_{TQF}) range from 0.0002 to 0.1 s in standard TQF experiments and from 0.0002 to 0.041 s for TQF imaging experiments. In order to make a reasonable comparison between 1D zg and 1D TQF for full-tube PSA in NaCl, the ratio of the number of scans of 1D TQF to 1D zg is

17.14 (Figure 4A in the main text), while the 1D imaging spectrum and 1D TQF imaging spectrum, the ratio of the number of scans of 1D TQF imaging to 1D imaging for half-filled PSA was adjusted to 11.25, while for full-tube PSA is 3.75 (Figure 3B in the main text). The number of scans of TQF relaxation rate measurements is 12 (Figure 4B in the main text).

Section III: Deconvolution of ^{23}Na 1D zg NMR spectra with half-filled PSA hydrogels

The 1D zg spectra of half filled PSA in NaCl or multisalt solutions were deconvoluted using Lorentzian-Gaussian fitting to resolve the overlapping resonances (Figure S2). A broad peak at the chemical shift from 0.30 to 0.34 ppm and a sharp peak $\sim$0.20 ppm. The broad peak originated in the region because Na^+ ions within the hydrogel matrix experienced restricted mobility with stronger quadrupolar interactions, which results in line broadening. However, it should be noted that the sample in NaCl+$MgCl_2$ with controlled concentration did not show a clear deconvoluted broad peak, which suggested the bound Na^+ ions are significantly reduced due to the destabilization of the hydrogel matrix caused by Mg^{2+} ions.

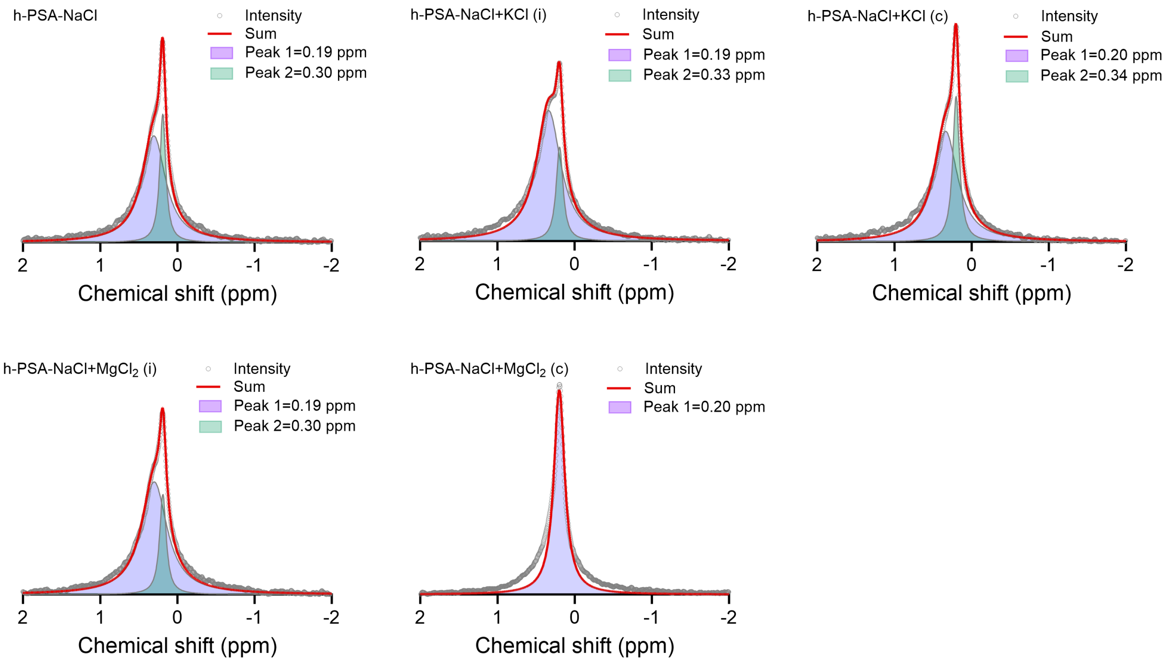

Figure S2. The 1D zg spectra of half-filled PSA in NaCl solution and multi salt solutions at room temperatures. The peak deconvolution was analyzed using MestreNova.

Section IV: Investigating potential ^{23}Na ionic exchange dynamics

In the CPMG experiment, the apparent transverse relaxation rate $R_{2,\text{eff}}$ depends on the echo delay (τ_{echo}) and $R_{2,\text{eff}}$ changes with τ_{echo} based on the Block-McConnell equation for a two site exchange system.[S1–S3] When the ionic interactions in the system are negligible, the $R_{2,\text{eff}}$ remains constant with the change of τ_{echo}. However, if there is a presence of ionic interactions, a dispersion in $R_{2,\text{eff}}$ can be observed as τ_{echo} increases. To identify whether ionic exchange interactions between Na^+ ions and electrolyte groups within hydrogels affect relaxation rates, we performed standard CPMG experiments with interpulse delay at 0.001 s, 0.002 s, 0.003 s and 0.006 s. The CPMG relaxation delay (τ_{CPMG}) was set from 0.012 to 0.0192 s. We performed the CPMG experiments on half-filled PSA in NaCl at room temperature, and the R_2 results did not show a significant change as τ_{echo} increases (Figure

S3).

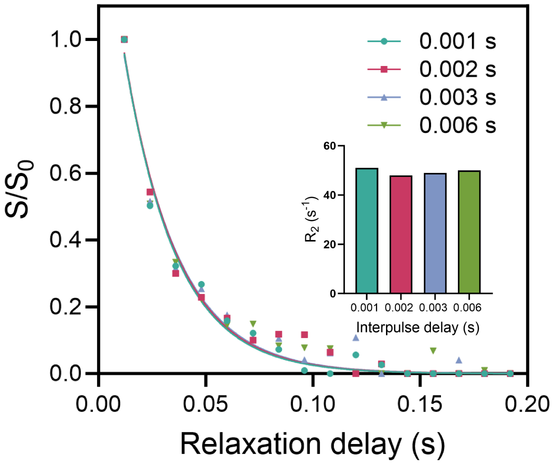

Figure S3. The CPMG measurements of half-filled PSA in NaCl at different interpulse delay ($\frac{\tau_{echo}}{2}$) at room temperature. The number of echos was adjusted to ensure the relaxation delay remains the same with the change of interpulse delay.

Section V: Temperature trend and reproducibility of 1D imaging profiles

The 1D ^{23}Na imaging profiles of half-filled PSA in NaCl measured from 298 to 318 K are shown in the Figure S4, and the results profiles in Section VI were normalized by 17.1 mM NaCl solution except the one for NaCl solution only (Figure S8). We can directly compare the signal integral changes of the gel region and supernatant phase as the temperature increases. The signal integral of the gel region increased significantly while the supernatant phase did not show clear changes. Moreover, for the 1D imaging profile of half-filled PSA in NaCl+$MgCl_2$ solution with controlled concentration, a repeat experiment was performed by preparing the sample separately and collecting data under the same conditions (Figure S5). Prior to Fourier transformation, a Sine Square II apodization function was applied to all profiles, with a shift parameter of 0.0% and an end point of 11.0% of the FID with the line broadening of 1.

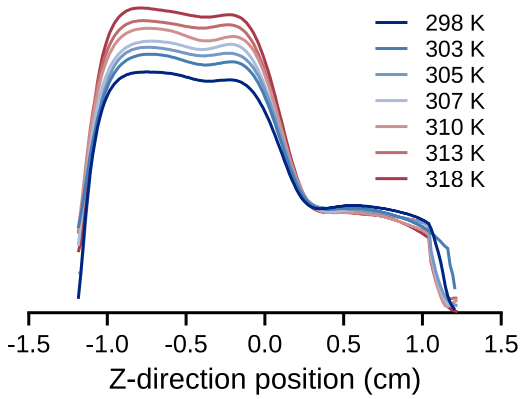

Figure S4. The 1D ^{23}Na imaging profiles of half-filled PSA in NaCl at the temperature from 298 to 318 K with the number of scans equal to 128.

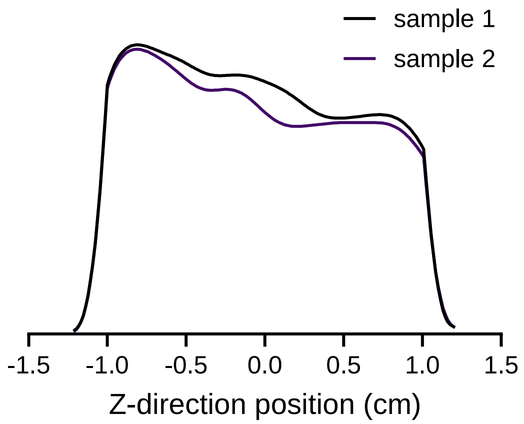

Figure S5. The repeated 1D ^{23}Na imaging experiments for half-filled PSA in NaCl+$MgCl_2$ solution with both salt ions are 17.1 mM at room temperature. The number of scans is 128. The signal intensity was divided by 17.1 mM NaCl solution. Two samples were prepared separately and measured at the same conditions.

In addition to measure the integral changes of each phase, we also determined the volume changes caused by gel expansion in the time-process 1D ^{23}Na imaging measurements. We run an additional 1D imaging experiment by comparing the volume differences between the sample at the swelling time of 0.25 hours and after 1 month, and an approximate 0.2 cm z-direction extension or 0.039 cm^3 volume expansion was observed in the gel region within the NMR detection region (Figure S6).

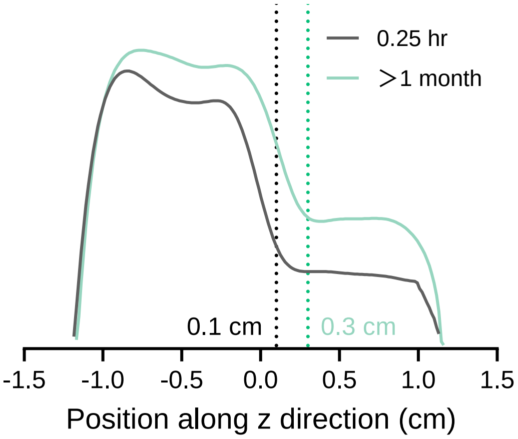

Figure S6. The 1D ^{23}Na imaging experiments for half-filled PSA in NaCl solution at the swelling time of 0.25 hours and more than one month. The measurements were performed under room temperature. The number of scans is 128. The signal intensity was divided by 17.1 mM NaCl solution.

The R_2 results of the hydrogel phase and the supernatant phase were measured using CPMG imaging as the swelling was processed (Figure S7). The hydrogel phase showed a clear decrease as the swelling time increased, while the change of R_2 in the supernatant phase was not obvious.

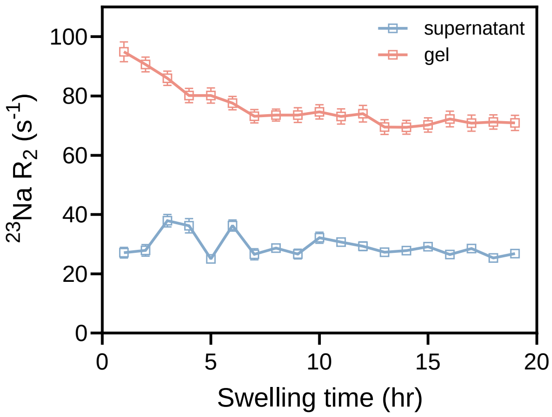

Figure S7. The ^{23}Na CPMG imaging experiments for half-filled PSA in NaCl solution during the swelling process. The R_2 of the hydrogel phase (red) and supernatant phase (blue) was plotted as the function of swelling time. The measurements were performed under room temperature.

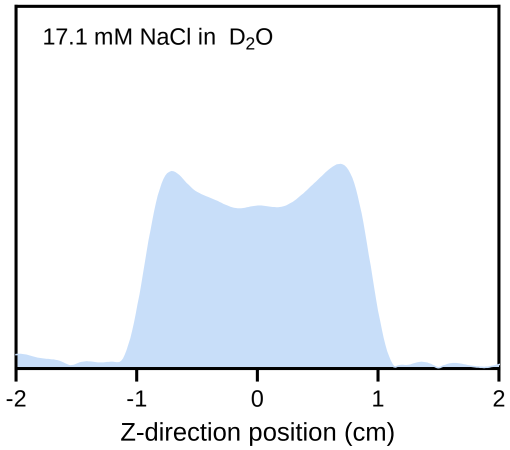

Figure S8. The ^{23}Na 1D imaging profile of 17.1 mM NaCl solution (D_2O) at room temperature. The number of scan is 128.

References

(S1) McConnell, H. M. Reaction Rates by Nuclear Magnetic Resonance. *J. Chem. Phys.* **1958**, *28*, 430–431.

(S2) Carver, J. P.; Richards, R. E. A General Two-Site Solution for the Chemical Exchange Produced Dependence of $T2$ upon the Carr-Purcell Pulse Separation. *J. Magn. Reson.* **1972**, *6*, 89–105.

(S3) Palmer, A. G. Chemical Exchange in Biomacromolecules: Past, Present, and Future. *J. Magn. Reson.* **2014**, *241*, 3–17.
